# American Academy of Dental Science

**Published:** 1903-01

**Authors:** 

**Affiliations:** American Academy of Dental Science


### Reports of Society Meetings.

### AMERICAN ACADEMY OF DENTAL SCIENCE.

THE regular monthly meeting of the Academy was held at Young's Hotel, Boston, on Wednesday evening, April 2, 1902, at six o'clock, President Bradley in the chair.

*President Bradley.*—An opportunity will be given to the Fellows of the Academy to present specimens of office practice at this time.

*Dr. Hopkins.*—This is not an incident of office practice. It is something that I have been using as a styptic, and I thought others might possibly have use for it. It is the active principle of the suprarenal gland in a normal salt solution, one to a thousand, and as a styptic it seems to be of considerable value in those cases where a rubber dam cannot be supplied; and where you are working down to the gum margin it really seems to be effective without scarifying the gum in any way, or leaving any sort of a slough. I have had a good deal of difficulty in getting any literature on the subject. I have written to the manufacturers, and have not heard from them as yet, and I cannot get any information here, so that I cannot tell in what liberal quantities this may be used. So far I have been a little cautious in protecting the mouth, but as the use of the suprarenal gland is that of a cardiac stimulant, it is likely that it will not do harm if used in any moderate quantities. Adrenalin is the name of the preparation, and it is prepared by Parke, Davis & Co.

*Dr. Ames.*—I have something here that may be of interest to the Fellows. It is a set of teeth made entirely of porcelain, that a lady had made by Dr. Scarboro, of New Jersey, and she has worn it twenty-six years. I will pass it around.

*President Bradley.*—Is this on a porcelain base?

*Dr. Ames.*—It is all porcelain. It has been worn twenty-six years.

*Dr. Andrews.*—I have a case for which I would like advice. A lady patient came to me and stated that her sister, an invalid, has been suffering at times from scalding pains in her mouth. The sensation is as though the mouth was burning. I have not seen the patient. The lady who called asked me if it were possible to help her sister. She has been under medical care without any relief whatever. They tell her it is probably a nervous trouble. I asked my patient if she would kindly write her sister's symptoms, and she wrote the following, which, with your permission, I will read.

"The trouble in my sister's mouth began as far back as 1857, but was not then nearly as painful as of late years, and now not equally painful at all times, sympathizing, apparently, with her nervous condition. The tongue and gums are often very red, sore and sensitive. The sensation is of red-hot coals in the mouth.

Acids, as in certain fruits, seem to aggravate the stinging and burning sensations, which my sister often says is enough to make her ill of itself."

I shall be glad to know of a remedy. I had a patient, an old lady, some years ago, who had trouble of this nature. There was very little that I could do to relieve it. Her symptoms were reddish, swollen gums, and a sensation as though they were burning. What I would like to inquire is, have any of our members had such cases, and, if so, what treatment was pursued?

*Dr. Cooke.*—Are the teeth present?

*Dr. Andrews.*—Some of them.

*President Bradley.*—Is there any other suggestion in regard to this matter?

*Dr. Andrews.*—Dr. Fillebrown said he had a case that he cured by hypnotism.

*Dr. Cooke.*—On that line, I do not know whether it would do any good in this case or not, but I had a patient in to-day whom I see frequently in regard to pyorrhœa trouble. She had been using during the last year precipitated chalk to pack around her teeth at night, and it has kept the trouble down a great deal, and the mouth seems to be in a great deal healthier condition than when that was not used.

I want to pass around a few models. This shows the bad results from extraction of the second temporary molars, and the six-year molars have moved forward. And also the temporary cuspids have been taken out.

*President Bradley.*—Is there any other item of interest that could be presented at this time?

*Dr. Cooke.*—I have a crown here, Mr. President, that I am going to talk about by and by, and I will pass it around. It was worn five years. A clasp went around this and held a plate up. It had a post crown on it before. It is a very poorly fitting band, and it is surprising that it stayed in a mouth. I made it.

*President Bradley.*—I believe Dr. Hopkins has an article that he would be willing to read to the Academy at this time, if the Fellows would please give it their attention.

*Dr. Hopkins.*—This is a very long and interesting article, on the subject of the variation in the power and composition of saliva, which was published in the *American Journal of Physiology* in 1898, and the summary may be interesting, and will only take two minutes to read. (Reads.)

*Dr. Werner.*—I think that is very interesting, and I want to ask the definition of amylolytic.

*Dr. Hopkins.*—Power to digest starch.

*President Bradley.*—I have the pleasure of introducing to you Dr. Horatio C. Meriam, D.M.D., who will read his paper on "Some Uses for English Tube-Teeth."

(For Dr. Meriam's paper, see page 1.)

*President Bradley.*—Dr. Meriam is going to pass around the specimens that he has spoken of. We have all been extremely interested in the complete demonstration of the subject which Dr. Meriam has presented to us this evening. It is, I am sure, prolific of discussion, which is to be opened by Dr. Cooke, according to the programme on the announcement card.

DISCUSSION.

*Dr. Cooke.*—Mr. President, I cannot expect to compete with this exhibit, as I think it will attract attention and I shall not be heard.

I have used these English tube-teeth in a good many ways. I think there is one decided objection to them, and that is the material out of which they are made. It is very nice to be able to grind them down and polish them up, but the material is so soft that they are not strong enough for the cases in which I want them, and the teeth are very liable to split. If we could have a tooth made the same way, but hard, I think it would be a great deal better.

Then again, Dr. Meriam did not speak of their use in plates. I had a patient who came from England, where they have used this type of tooth very successfully. This was a partial case where the molars and bicuspids were put on by means of the tube-teeth on a plate. I had one case which was not made of gold, but of dental alloy, which is a good deal cheaper than gold. I had one case that I sent down to one of the dental laboratories to have a tooth ground on, and they kept a man on it all day, and then sent it back partly done, saying they did not want another job of that kind. It is a very easy thing to grind the teeth in a case of that sort if you only go at it right.

This was a case of a lower partial plate, there being two bicuspids and two molars, and this would be a cross-section of this portion (illustrating on the board). When you come to put on your teeth, grind them first about where you want them to go and then put your pin in the plate, and it is quite easy to grind the teeth in place. But if you try to do it the other way, it is a long, hard job and liable to be unsatisfactory.

Whether you make a tooth of your English tube-teeth, or whether you make a case of your regular carved tooth and solder your backings on, as we ordinarily do, I should use sulphur for filling around between this part and the gold. It will make a clean case. We all know when teeth are cut off of a plate there is a lot of material between the teeth and the plate. Pour your sulphur all around into your joint when the tooth is ground to fit the plate. That is one of the best uses of sulphur, and it does very nicely in these cases.

Another thing, these teeth will wear well on a plate and will not crack, where, when you come to a crown it is entirely different. For the front teeth I do not like them,—*i.e.*, the upper six teeth, the anterior teeth; I do not care for the shape of them. I have used them for bicuspids, and in the case I passed around they look very well in the mouth. I could not tell how long that root was, and the patient had had a crown, and this clasp went around the tooth and held it in position. That is an advantage where you use a clasp. Where you put an all metal crown on and then a clasp, you are going to have wearing of your crown.

Then, in regard to the use of a post, I do not think it is necessary to use a post in these cases. I simply put on a band with a top to it, and do not use a post, and they do not come off. I have had one or two cases of fracture. I remember one or two cases where the sulphur has failed,—*i.e.*, where the tooth has loosened from the post on account of the sulphur giving way. It is a very easy thing to put those on. I used to take the top of a tin box, or something of that sort, to put the sulphur in, but it soon turned very dark; and then I procured a little porcelain saucer, and the sulphur retained its color. In getting the relations of the crowns, for instance, I put gold right across the end of the band, try my crown in the mouth to see just where it wants to go, and take an impression of that crown in the general relation. In some cases your crown will not be large enough. In those cases, after you have fitted your crown, take a little of the low-fusing porcelain and make it fit, and round it down in nice shape.

It is possible to take a crown, scratching it where the post should go, put your post on, and solder your post. It is a good deal easier to carry your post directly through the top of the gold cap and then solder it, and cut off the top of your post if you do not wish to use the post in the root.

Dr. Meriam had a space left between the porcelain and the root. I think the more space you have between the porcelain and the end of the root, the worse you are off. I think in using the cement it is just as it is in the inlay,—the less cement you have between the porcelain and the root the better. If you can have a tight joint there, and have the least possible quantity of cement, so much the better. Also the less space you have around your post in fitting the root-canals the better.

As for the grinding out on the end of the headed pin, I do not think that amounts to much. The more you grind, the more you weaken your porcelain, and I do not believe a patient can tell whether it is gold or the regular metal of the pin.

As to the thickness of the band, I have used the ordinary 29, 30, or 31 coin gold rolled down, and I have not had any trouble with the crowns failing on that account. For the grinding surface, of course, it is a fact that you get a good deal better grinding surface with the porcelain than with gold.

*President Bradley.*—The subject is still open for discussion, and we should be glad to hear from any others, our guests as well, or any other member of the Academy.

*Dr. Werner.*—Mr. President, the subject is so large, so many things presented, if I say anything it will be in regard to Dr. Meriam's extension crowns. Since he has shown before the Academy several practical cases, I have tried it, and to me it is a satisfactory and practical operation.

I do believe in having the teeth carved. I never put one on yet where I did not have the tooth carved, with two pins and a saddle, and they have been so far very satisfactory; they are clean. I do not see the slightest objection to the saddle. I do not think it is necessary to mark on the black-board what I mean by the saddle, but perhaps it is just as well.

The first case I ever did after Dr. Meriam's style was a lateral incisor, and I went back for anchorage to the first molar; the second bicuspid, the first bicuspid, and the cuspid were in place. This was not a carved tooth, but a rubber-plate tooth, and had no saddle. Many times I prefer a rubber-plate tooth to a gold-plate tooth. That molar was not trimmed, not even a polishing strip or sand-paper disk, but was thoroughly clean. I took a plaster impression, fitted the band on the buccal side about one-sixth the width of the crown, and on the palatal side covered the crown from the bulging part to the cutting edge. The band on the inside was wide; on the outside it was rounded, thick, and narrow.

I do not like bands near the gum. There is a good deal of spring where the extension arm is from the molar to the lateral incisor, and it should be of platinized gold, half-round, and resting firmly on the gum.

My second case was a first bicuspid. I also had the first molar as anchorage. The bicuspid crown was carved with a distinct saddle, and that went on without the least trimming of the first molar tooth; the band was driven on, and it fitted. This, which I call the Meriam extension crown, is a very practical application.

We should all remember the necessity of contouring, whatever crown or whatever kind or method of crown we use. I think it essential to have a proper contour. If you have that properly done, a good many minor things, that will afterwards turn out large things, will be avoided, such as cleanliness, comfort, and proper enunciation. I think proper contour in all crowns a very essential thing.

Adjourned.

Charles H. Taft,
*Editor American Academy of Dental Science.*

